# Cytokine/chemokine profiles in people with recent infection by *Mycobacterium tuberculosis*

**DOI:** 10.3389/fimmu.2023.1129398

**Published:** 2023-05-16

**Authors:** Mariana Herrera, Yoav Keynan, Lucelly Lopez, Diana Marín, Lázaro Vélez, Paul J. McLaren, Zulma Vanessa Rueda

**Affiliations:** ^1^ Epidemiology Doctorate, Facultad Nacional de Salud Pública, Universidad de Antioquia, Medellín, Colombia; ^2^ Department of Medical Microbiology & Infectious Diseases, University of Manitoba, Winnipeg, MB, Canada; ^3^ Departments of Internal Medicine and Community Health Sciences, University of Manitoba, Winnipeg, MB, Canada; ^4^ Facultad de Medicina, Universidad Pontificia Bolivariana, Medellín, Colombia; ^5^ Grupo de Investigación en Salud Pública, Universidad Pontificia Bolivariana, Medellín, Colombia; ^6^ Grupo Investigador de Problemas en Enfermedades Infecciosas (GRIPE), Facultad de Medicina, Universidad de Antioquia, Medellín, Colombia; ^7^ JC Wilt Infectious Diseases Research Centre, Public Health Agency of Canada, Winnipeg, MB, Canada

**Keywords:** tuberculosis infection, TST conversion, *Mycobacterium tuberculosis*, cytokines, chemokines, tuberculosis

## Abstract

**Introduction:**

The risk of progression to tuberculosis disease is highest within the first year after *M. tuberculosis* infection (TBI). We hypothesize that people with newly acquired TBI have a unique cytokine/chemokine profile that could be used as a potential biomarker.

**Methods:**

We evaluated socio-demographic variables and 18 cytokines/chemokines in plasma samples from a cohort of people deprived of liberty (PDL) in two Colombian prisons: 47 people diagnosed with pulmonary TB, 24 with new TBI, and 47 non-infected individuals. We performed a multinomial regression to identify the immune parameters that differentiate the groups.

**Results:**

The concentration of immune parameters changed over time and was affected by the time of incarceration. The concentration of sCD14, IL-18 and IP-10 differed between individuals with new TBI and short and long times of incarceration. Among people with short incarceration, high concentrations of MIP-3α were associated with a higher risk of a new TBI, and higher concentrations of Eotaxin were associated with a lower risk of a new TBI. Higher concentrations of sCD14 and TNF-α were associated with a higher risk of TB disease, and higher concentrations of IL-18 and MCP-1 were associated with a lower risk of TB disease.

**Conclusions:**

There were cytokines/chemokines associated with new TBI and TB disease. However, the concentration of immune mediators varies by the time of incarceration among people with new TBI. Further studies should evaluate the changes of these and other cytokines/chemokines over time to understand the immune mechanisms across the spectrum of TB.

## 1 Introduction

To reduce the burden of Tuberculosis (TB) worldwide, two key components will be necessary: 1) prevention of new TB infection (TBI) and 2) prevention of the progression from TBI to TB disease (1). The global prevalence of TBI is 24.8% (95% CI: 19.7-30.0%) and 21.2% (95% CI: 17.9-24.4%) based on the results of the interferon-gamma release assays (IGRAs) and tuberculin skin test (TST), respectively (2). Prevalence among people who are at higher risk for TBI, including people deprived of liberty (PDL), is generally higher than the community at large in the same geographic location and can be as high as 88.8% (3).

Control of the progression to TB disease relies on accurate TBI diagnosis. However, this is one of the significant challenges in the path toward TB elimination. The main limitations of current tests (TST and IGRAs) are their inability to distinguish between TBI and TB disease or to predict TB progression to active disease (4–8). Despite numerous articles reporting immune markers associated with TBI and TB disease, there is high heterogeneity in the design, participant selection, sample processing (including the stimulation protocols), measured markers and analysis that limits comparing study results (9).

TB disease occurs most frequently among people newly infected with TB, mainly within the first year of acquiring mycobacterial infection (10). Identifying and prioritizing those with new TBI within the first year of mycobacterial infection to offer TBI treatment could be an effective measure to decrease TB transmission.

This study focused on incarcerated population because of the high risk of exposure to TB and progress to TB disease (11). We analyzed data and samples collected from two prisons where we have previously shown high rates of TB infection and disease (12, 13). This study aimed to determine the plasma concentration of 18 cytokines/chemokines associated with the presence of new *Mycobacterium tuberculosis* (MTB) infection and compare it with the concentration among people with pulmonary TB diagnosis and exposed but uninfected people.

## 2 Methods

### 2.1 Ethics statement

Approval for the study was obtained from the Ethics Committees of the Universidad Pontificia Bolivariana and the University of Manitoba. The Instituto Nacional Penitenciario y Carcelario (INPEC) and the director of each prison approved the project. In all cases, written consent forms were explained and signed in the presence of two witnesses (always people deprived of liberty -PDL).

### 2.2 Study design, settings, and population

This cohort study was conducted in Colombia between September 2016 and December 2018 in two medium and high-security men’s prisons. According to the inclusion criteria, the cohort included 124 PDL with a negative two-step TST at enrolment (**Supplementary Material 1**). Complete information related to the protocol, procedures, eligibility, recruitment, follow-up and epidemiological data for this cohort study has been published elsewhere (13).

During the cohort study, the TBI incidence rates varied between 2,402.88 cases per 100,000 person-months (95% CI 1,364.62 - 4,231.10) in PDL with short time of incarceration to 419.66 issues per 100,000 person-months (95% CI 225.80 - 779.95) in individuals with a long time of incarceration (13). For this reason, the cohort was divided into two subgroups: 64 “PDL with short incarceration” for those who were enrolled in follow-up upon incarceration or within the first three months of incarceration, and 60 “PDL with long incarceration” for people who started their follow-up after one year or more of imprisonment.

The primary outcome in the cohort was a new TBI (documented TST conversion). In the end, there were 25 new TBIs among 124 people with a negative two-step TST at enrolment. Thirteen out of 25 were people with short incarceration, and 12/25 new TBIs were people with long incarceration. Ninety-nine individuals remained TST negative after the follow-up. **Supplementary Material 1** has information about the new TBI diagnosis criteria.

In addition, we included 51 of 88 (57.95%) people with a new pulmonary TB diagnosis during the study period. The prison healthcare system performed the microbiological diagnoses, and we also collected sputum samples to confirm *Mycobacterium tuberculosis*. People were invited to participate in the study after the TB diagnosis. **Supplementary Material 1** describes the eligibility criteria and the TB diagnosis.

### 2.3 Procedures

#### 2.3.1 Data collection

The socio-demographic data were collected from all individuals at baseline: age; history and time of prior incarceration; use of drugs (inhaled, injected, or smoked), smoking, and alcohol consumption; comorbidities (chronic obstructive pulmonary disease, diabetes, chronic kidney disease, HIV, and any other immunosuppressive condition); contact with a person diagnosed with TB disease (outside and inside the prison); history of prior TB, including date of the last episode, and outcome; weight and height. To determine previous exposure to the BCG vaccine, the field team sought the presence of the BCG scar.

#### 2.3.2 Blood sample collection

All PDL included in the study provided blood samples at baseline and every three months during their follow-up. The samples were collected in sodium heparin tubes, and plasma was separated and stored at -80°C until processing.

**Figure 1:**
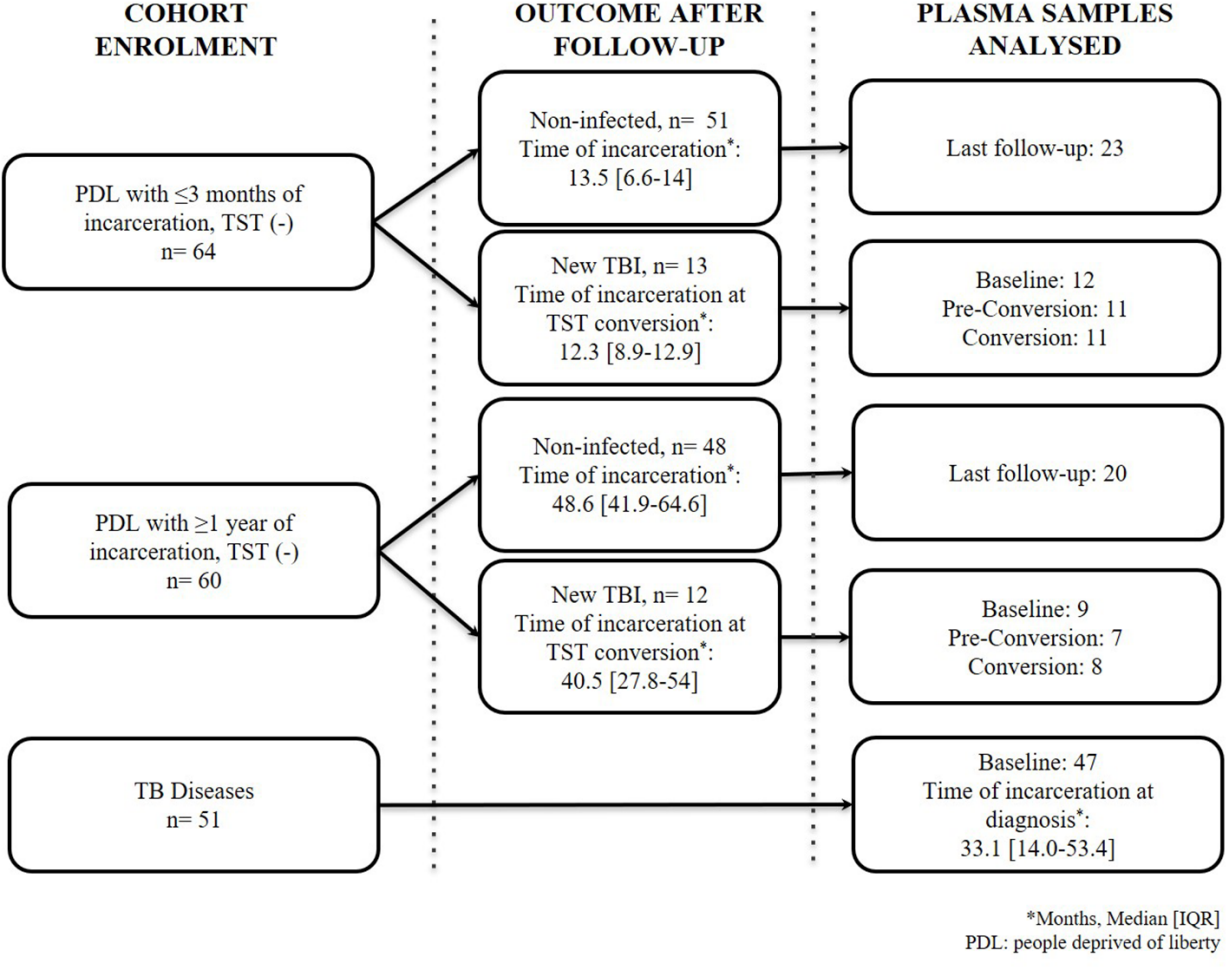
Flow chart of people included in the study. TB: tuberculosis; TST: tuberculin skin test. New TBI [people with negative two-step TST that became positive during follow-up were divided into TBI with short incarceration (they had ≤3 months of incarceration at enrolment) and TBI with long incarceration (they had ≥1 year of incarceration at enrolment)].

We processed 148 samples as follows: a) 58 samples of PDL with a new TBI (21 samples at baseline, 18 at pre-conversion [3 months before TST conversion], and 19 at the time of TST conversion); b) 43 samples of PDL of the last follow–up available among people that remain TST negative and had the longest follow-ups; c) and 47 samples at baseline of people diagnosed with TB disease (**Figure 1**). We excluded 4/51 participants diagnosed with TB because 3 had HIV co-infection, and one did not have a plasma sample at baseline.

#### 2.3.3 Cytokines and chemokine selection and detection

The cytokines/chemokines quantified in the study were selected based on: 1) a systematic review we conducted to identify the relevant cytokines/chemokines associated with TBI (14), 2) published reviews about the pathophysiology of *M. tuberculosis* infection (15–17), 3) results from animal models (guinea pig, macaques and mice) (15, 16, 18–20), 4) immune response to intracellular bacteria (21, 22), 5) *M. tuberculosis* pathway (23) (available at: http://www.genome.jp/kegg/pathway.html) and 6) our previous results among people diagnosed with TB disease (24).

Commercial multiplex and single bead-based fluorescent assay kits were used to quantify 18 cytokines/chemokines of interest from plasma samples as follows: Macrophage Inflammatory Protein 3α (MIP-3α/CCL20), Human Cytokine/Chemokine magnetic Panel III, Milliplex^®^ Map kit); Interleukin 18 (IL-18), Human IL-18 Singleplex Magnetic Bead kit, Milliplex^®^ Map kit; soluble CD14 (sCD14), Human Cardiovascular Disease (CVD) Panel 6 Magnetic Bead kit, Milliplex^®^ Map kit; Eotaxin 1 (CCL11), Interferon gamma (INF-γ), Interleukin 5 (IL-5), Interleukin 6 (IL-6), Interleukin 10 (IL-10), Interleukin IL-12 p40 homodimer (IL-12[p40]), Interleukin 13 (IL-13), Interleukin 15 (IL-15), Interleukin (IL-17), Interleukin-1 receptor antagonist (IL-1RA), human interferon-inducible protein 10 (CXCL10/IP-10), monocyte chemoattractant protein-1 (CCL2/MCP-1), macrophage inflammatory protein 1α (CCL3/MIP-1α), macrophage inflammatory protein 1β (CCL4/MIP-1β), Tumor necrosis factor alpha (TNF-α), Human Cytokine/Chemokine magnetic Bead Panel, Milliplex^®^ Map kit, Millipore Corporation, Billeria, MA, USA.

The assays were performed according to the manufacturer’s instructions, using 25 µl of plasma per sample (5 µl to run the sCD14 assay) and overnight incubation. Standards were reconstituted and serially diluted to generate standard curves. Two controls with low and high concentrations, provided by the commercial kit, were included in each assay and considered positive controls for the experiment. Results were analyzed in the BioPlex-200 instrument (Bio-Rad, Mississauga, Canada), reported as mean fluorescence intensity, and converted to pg/ml or ng/ml using the BioPlex^®^ Manager version 6.0 (Bio-Rad, Mississauga, ON).

To control for potential biases, all specimens were analyzed, blinded to the clinical status, and longitudinal samples were performed by the same person and analyzed on the same plate. Samples with values outside the standard curve range were assigned a value of one-half of the lower limit of detection (LOD divided by 2) in pg/mL.

### 2.4 Analysis

We used descriptive statistics (median [IQR] and n [%]) to report cytokines/chemokines and socio-demographic variables, and chi-squared and Kruskal Wallis tests to evaluate differences between groups. The primary outcome of this study was a new TBI stratified by short and long incarceration.

In individuals with new TBI, we compared the concentration of the immune mediators at baseline, three months before the TST conversion (pre-conversion point) and TST conversion. Wilcoxon test was used to compare the changes in cytokines/chemokines over time. In addition, we used the Mann-Whitney test to compare each follow-up between individuals with a new TBI with short and long times of incarceration.

Then, we compared plasma concentration of cytokines/chemokines in people with new TBI with short and long incarceration to non-infected individuals and people diagnosed with pulmonary TB using the Kruskal-Wallis test. We used multinomial logistic regression to determine the association between each cytokine/chemokine and new TBI with short or long incarceration compared to TB disease and non-infected groups. All cytokines/chemokines were log-transformed to adjust for skewness before the multivariable analysis. Variables included in the final regression model were selected using the biological plausibility criteria, a manual backward elimination method. We adjusted the model by age, BCG scar, contact with a person diagnosed with TB and drug use. All models were adjusted by cluster effect (15 different courtyards in the two prisons).

All analyses were done using STATA^®^ version 14. A two-tailed p-value <0.05 was considered significant. Considering the multiple comparisons were made when we evaluated the cytokine concentration between the groups, a *p-*value <0.01 was considered significant, applying the Bonferroni correction.

## 3 Results

### 3.1 Study participants

The median time of TST conversion (new TBI) among the short incarceration group was 12.3 months, and in the new TBI with long incarceration group was 40.5 months (**Figure 1**). The majority of new TBIs occurred in one prison (75%).

There was no significant difference between groups regarding the mean age (**Table 1**). Ten individuals in the study had a body mass index ≤18.5 kg/m^2^, and 9 of them had a TB diagnosis. Individuals diagnosed with TB reported higher consumption of smoked drugs (61.7%) and inhaled drugs (34%) compared to the other groups (p ≤ 0.001). There was no difference in the frequency of alcohol consumption and tobacco use (p>0.051) (**Table 1**). Having contact with a person diagnosed with TB was reported in 8.3% of the new TBI group, 34% of people diagnosed with TB, and 19.2% in the non-infected group. **Table 1** reports socio-demographic information at baseline.

**Table 1:** Baseline characteristics of study participants diagnosed with a new TBI (with short and long incarceration), TB disease, and non-infected people.

Variable	Not infected (n=47) n (%)	New TBI, short incarceration (n=11) n (%)	New TBI, long incarceration (n=13) n (%)	TB disease (n=47) n (%)	*p-value*
Age, years, median [IQR]	34 [30-44]	33 [30-39]	31 [25-56]	32 [26-37]	0.201
Time of current incarceration, months, median [IQR]	11.2 [1.6-39.4]	12.3 [8.9-12.9]	40.5 [27.8-54]	33.1 [14-53.4]	**0.001**
BMI ≤18.5	1 (2.2)	0	0	9 (19.1)	**0.013**
Prison					**<0.001**
Prison One	22 (46.8)	7 (63.6)	11 (84.6)	47 (100.0)	
Prison Two	25 (53.2)	4 (36.7)	2 (15.4)	0	
Comorbidities	9 (19.1)	0	4 (30.7)	9 (19.1)	0.284
COPD	3 (6.4)	0	1 (7.7)	3 (6.4)	0.849
Diabetes mellitus	1 (2.1)	0	2 (15.4)	2 (4.3)	0.173
Psychiatric illness	3 (6.4)	0	1 (7.7)	0	0.256
Others	3 (6.4)	1 (9.1)	0	4 (8.5)	0.736
Inhaled drug use					**0.001**
Never	32 (68.1)	6 (54.5)	9 (69.2)	14 (30.0)	
Past	11 (23.4)	5 (45.5)	3 (23.1)	16 (34.0)	
Current	4 (8.51)	0	1 (4.17)	17 (34.0)	
Smoked drug use					**<0.001**
Never	28 (59.6)	7 (63.6)	7 (53.8)	7 (14.9)	
Past	11 (23.4)	2 (18.2)	3 (23.1)	11 (23.4)	
Current	8 (17.0)	2 (18.2)	3 (23.1)	29 (61.7)	
Tobacco consumption					0.294
Never	18 (38.3)	6 (54.5)	3 (23.1)	11 (23.4)	
Past	12 (25.5)	1 (9.1)	4 (30.7)	10 (21.3)	
Current	17 (36.2)	4 (36.4)	6 (46.1)	26 (55.3)	
Alcohol use					0.051
Never	12 (25.5)	3 (27.4)	1 (7.7)	8 (17.0)	
Past	22 (46.8)	7 (63.6)	8 (61.5)	17 (36.1)	
Current	12 (25.5)	0	3 (23.1)	22 (46.8)	
Occasional	1 (2.13)	1 (9.1)	1 (7.7)	0	
History of contact with a TB case	9 (19.1)	1 (9.1)	1 (7.7)	16 (34.0)	0.087
Contact with prisoner	7 (14.9)	1 (9.1)	1 (7.7)	14 (29.8)	
Contact with relative	2 (4.3)	0	0	2 (4.1)	
BCG Scar	45 (95.7)	8 (72.2)	8 (61.5)	41 (87.2)	**0.007**

IQR, interquartile range; TB, tuberculosis; COPD, Chronic obstructive pulmonary disease; BCG, Bacillus Calmette-Guerin. New TBI [people with negative two-step TST that became positive during follow-up were divided into TBI with short incarceration (they had ≤3 months of incarceration at enrolment) and TBI with long incarceration (they had ≥1 year of incarceration at enrolment)]. *p-value using the Kruskal-Wallis test for quantitative variables and Chi-square test for qualitative variables. Variables with statistical significance are shown in bold.

IL-10, IL-12, IL-13, IL-15, IL-17A, IL-1RA, IL-5, IL-6 and MIP-1α had more than 40% of the results below the lower detection limit. We did not see consistent trends of these results within and between groups or by the follow-up time. Therefore, these cytokines/chemokines were excluded from the analysis and results.

### 3.2 The concentration of cytokines/chemokines among the new TBI group is affected by the time of incarceration

**Supplementary Material S2** reports the median concentrations of all immune parameters at baseline, pre-conversion and TST conversion by short and long incarceration among the new TBI group.

**Figure 2:**
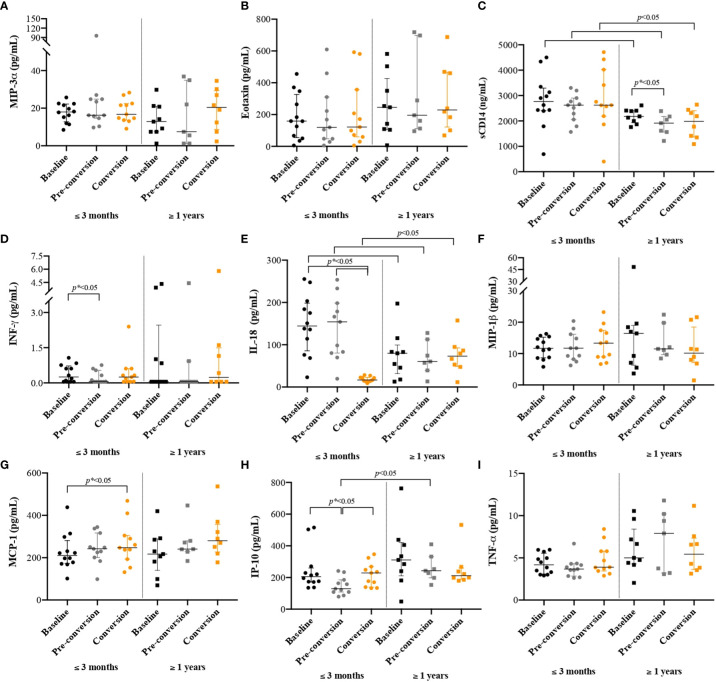
Cytokines/chemokines concentrations among people with new latent tuberculosis infection at baseline, pre-conversion, and TST conversion, by the time of incarceration. New TBI [people with negative two-step TST that became positive during follow-up were divided into new TBI with short incarceration (they had ≤3 months of incarceration at enrolment) and new TBI with long incarceration (they had ≥1 year of incarceration at enrolment)]. Values are reported in pg/ml for all cytokines/chemokines, except for sCD14 where results are reported in ng/ml. Boxplots are median and interquartile range. **(A)** MIP-3α; **(B)** Eotaxin; **(C)** sCD14; (ng/ml) **(D)** INF-γ; **(E)** IL-18; **(F)** MIP-1β; **(G)** MCP-1; **(H)** IP-10; **(I)** TNF-α.

Our results showed that there were no differences in the concentrations of MIP-3α (**Figure 2A**), Eotaxin (**Figure 2B**), INF-γ (**Figure 2D**) and MIP-1β (**Figure 2F**). We found higher concentrations of sCD14 at baseline compared to pre-conversion and TST conversion points; the change was more evident in PDL with long incarceration (**Figure 2C**). PDL with short incarceration showed increased plasma levels of IL-18 at baseline and pre-conversion, compared to the time of TST conversion (**Figure 2E**). MCP-1 showed an increased concentration over time, with a higher concentration at the time of TST conversion (**Figure 2G**). We also found that in PDL with short incarceration, IP-10 decreased three months before TST conversion (**Figure 2H**).

The concentrations of sCD14 (**Figure 2C**) and IL-18 (**Figure 2E**) were higher among people with new TBI and short incarceration compared to those with long incarceration (*p*< 0.05). The concentration of IP-10 (**Figure 2H**) was higher in people with long incarceration.

### 3.3 Cytokines/chemokines concentrations are different between groups

**Figure 3:**
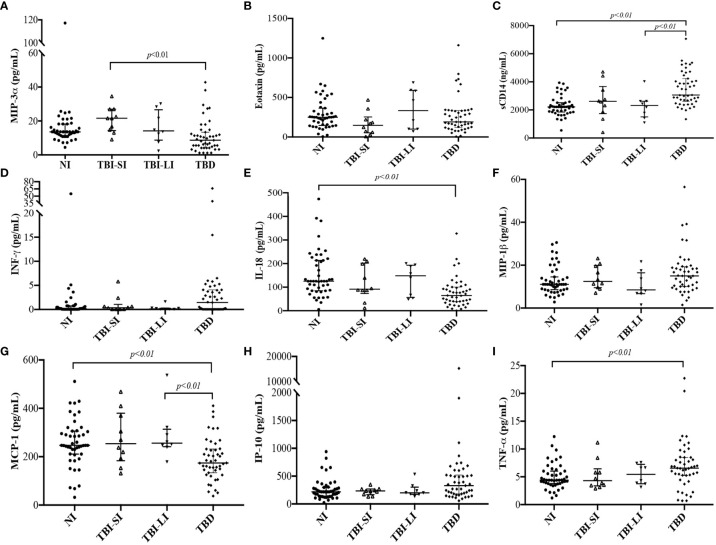
Immune parameters concentrations among people who converted the TST (new TBI with short incarceration [n= 11] and TBI with long incarceration [n= 13]), people diagnosed with TB disease (n=47), and non-infected people (n=47). New TBI [people with negative two-step TST that became positive during follow-up were divided into new TBI with short incarceration (they had ≤3 months of incarceration at enrolment) and new TBI with long incarceration (they had ≥1 year of incarceration at enrolment)]. **(A)** MIP-3α; **(B)** Eotaxin; **(C)** sCD14 (ng/ml); **(D)** INF-γ; **(E)** IL-18; **(F)** MIP-1β; **(G)** MCP-1; **(H)** IP-10; **(I)** TNF-α. Values reported in pg/ml for all cytokines, except for sCD14 reported in ng/ml. Boxplots are median and interquartile range. *p-value* using the Mann-Whitney U test and adjusted by multiple comparisons.

We found higher concentrations of sCD14 and TNF-α among people diagnosed with TB, MIP-3α among people with new TBI and short incarceration, and IL-18 and MCP-1 among people with new TBI and long incarceration (**Table 2**). We did not find differences in the concentration of Eotaxin, INF-γ, MIP-1β, and IP-10 among the groups (**Figures  3B, 3D, 3F, 3H**).

**Table 2:** The concentration cytokine/chemokine and differences between the groups (new TBI [with short and long incarceration], TB disease, and non-infected).

Cytokines/ Chemokines	Non-infected pg/ml (IQR)	New TBI and short incarceration pg/ml (IQR)	New TBI and long incarceration pg/ml (IQR)	TB disease pg/ml (IQR)	p-value*
sCD14 (ng/ml)	2213.8 (1851.9-2579.9)	2610.8 (1863.6-3413.0)	2314.9 (1596.8-2619.5)	3063.4 (2605.7-4233.1)	**0.0001**
MIP-3α	13.6 (10.9-18.2)	21.6 (14.8-26.9)	14.2 (9.9-24.9)	8.7 (4.1-13.4)	**0.0001**
IL-18	125.4 (84.5-220.8)	91.6 (86.3-198.9)	148.4 (59.9-191.1)	64.8 (38.1-100.5)	**0.0001**
Eotaxin	249.3 (141.3-375.9)	146.8 (59.8-218.8)	334.0 (100.4-587.2)	190.4 (118.3-333.4)	0.2421
INF-γ	0.19 (0.05-0.8)	0.4 (0.05-0.6)	0.12 (0.05-0.3)	1.44 (0.05-4.1)	0.1714
MIP-1β	11.1 (8.2-17.0)	12.4 (9,7-19.6)	8.5 (6.7-15.5)	15.0 (10.1-19.2)	0.1532
TNF-α	4.4 (3.7-6.3)	4.3 (3.5-5.8)	5.4 (3.8-7.0)	6.6 (5.2-8.5)	**0.0466**
IP-10	217.4 (133.2-303.6)	233.7 (177.1-267.5)	199.7 (174.8-280.2)	328.7 (179.7-518.6)	0.0842
MCP-1	246.9 (207.3-307.5)	253.9 (193.1-369.8)	256.0 (243.1-305.8)	174.2 (133.6-231.7)	**0.0006**

IQR: interquartile range; TB: tuberculosis; TBI: tuberculosis infection *p value using Kruskal-Wallis test. New TBI [people with negative two-step TST that became positive during follow-up were divided into TBI with short incarceration (they had ≤3 months of incarceration at enrolment) and TBI with long incarceration (they had ≥1 year of incarceration at enrolment)]. Variables with statistical significance are shown in bold.

The concentration of sCD14 was higher in the TB disease group compared to non-infected individuals and new TBI with long incarceration group (**Figure 3C**). TNF-α was higher in the TB disease group compared to non-infected individuals (**Figure 3I**). MIP-3α was higher in the new TBI with short incarceration compared to the TB disease group (**Figure 3A**). MCP-1 was higher in the new TBI with long incarceration compared to the TB disease group (**Figure 3G**). IL-18 (**Figure 3E**) and MCP-1 (**Figure 3G**) concentrations were lower in the TB disease group compared to non-infected individuals.

### 3.4 There are cytokines/chemokines associated with a new TBI and TB disease

In the multivariable analysis, only having a BCG scar was associated with decreased risk of a new TBI in people with short incarceration. The other socio-demographic variables (age, history of having contact with a person diagnosed with TB, and drug use) were not associated with new TBI or TB disease. Although having a BMI of less than 18 kg/m^2^ and alcohol consumption were significant factors, the multinomial model did not converge when we included those variables due to the low number of outcomes in the ‘yes’ category.

In the final multinomial model (**Table 3**), we identified that higher concentrations of MIP-3α were associated with a higher risk of a new TBI. The presence of a BCG scar and higher concentrations of Eotaxin were associated with a lower risk of a new TBI among people with short incarceration compared to non-infected individuals.

**Table 3:** Cytokines/chemokines associated with new TBI with short or long incarceration, TB disease compared to non-infected people in a multinomial regression model.

Cytokines/ Chemokines (Log transformed)	New TBI with short incarceration		New TBI with long incarceration		TB disease	
	cRR^+^ [95% CI]	aRR*^+^ [95% CI]	cRR^+^ [95% CI]	aRR*^+^ [95% CI]	cRR^+^ [95% CI]	aRR*^+^ [95% CI]
Non-infected	1 [Reference]	1 [Reference]	1 [Reference]	1 [Reference]	1 [Reference]	1 [Reference]
sCD14	1.17 [0.09-13.92]	2.62 [0.59-11.69]	0.87 [0.31-2.41]	0.76 [0.09-6.40]	24.17 [8.66-67.42]	**77.78 [7.60-796.16]**
MIP-3α	2.40 [1.47-3.93]	**7.46 [2.01-26.72]**	0.80 [0.29-2.18]	0.96 [0.32-2.93]	0.28 [0.11-0.66]	0.33 [0.09-1.19]
IL-18	0.55 [0.16-1.88]	0.31 [0.04-2.57]	0.86 [0.39-1.93]	1.00 [0.48-2.08]	0.30 [0.10-0.88]	**0.13 [0.02-0.91]**
Eotaxin	0.53 [0.35-0.83]	**0.35 [0.19-0.65]**	1.27 [0.56-2.90]	1.31 [0.60-2.84]	0.81 [0.53-1.25]	1.35 [0.61-2.98]
INF-γ	1.09 [0.76-1.56]	1.13 [0.60-2.13]	0.85 [0.52-1.39]	0.81 [0.35-1.87]	1.28 [1.05-1.56]	1.45 [0.91-2.30]
MIP-1β	1.56 [0.61-4.01]	6.87 [0.74-63.74]	0.49 [0.25-0.98]	0.50 [0.09-2.55]	1.78 [0.92-3.45]	2.91 [0.73-11.57]
TNF-α	1.10 [0.44-2.76]	0.27 [0.04-1.80]	1.34 [0.61-2.92]	2.53 [0.23-28.28]	2.00 [0.64-6.27]	**3.38 [1.01-11.24]**
IP-10	1.13 [0.72-1.77]	0.72 [0.17-2.95]	1.21 [0.65-2.27]	1.06 [0.16-6.80]	2.58 [1.51-4.42]	1.41 [0.29-6.97]
MCP-1	1.81 [0.35-9.40]	1.75 [0.10-29.26]	3.15 [1.74-5.69]	1.65 [0.30-8.91]	0.9 [0.15-0.55]	**0.03 [0.006-0.18]**
BCG scar	0.12 [0.01-1.04]	**0.04 [0.002-0.43]**	0.07 [0.01-0.57]	0.12 [0.003-5.67]	0.29 [0.05-1.52]	0.07 [0.003-1.32]
Age in years	0.98 [0.95-1.02]	0.95 [0.88-1.02]	1.02 [0.99-1.04]	1.01 [0.95-1.08]	0.98 [0.95-1.00]	0.98 [0.91-1.04]

cRR, crude relative risk *aRR: adjusted relative risk based on multinomial logistic regression analysis; TB, tuberculosis. New TBI [people with negative two-step TST that became positive during follow-up were divided into TBI with short incarceration (they had ≤3 months of incarceration at enrolment) and TBI with long incarceration (they had ≥1 year of incarceration at enrolment)]. All variables included in the multinomial regression model were selected through a manual backward elimination method. ^+^The bivariable and multivariate models are adjusted by cluster effect (15 courtyards). Variables with statistical significance are shown in bold.

Higher plasma concentrations of sCD14 and TNF-α were associated with an increased risk of TB disease. Finally, we found that higher concentrations of IL-18 and MCP-1 were associated with a lower risk of TB disease compared to non-infected individuals (**Table 3**).

## 4 Discussion

The main results of our study were: 1) the concentration of immune mediators in persons with a new TBI varies according to the time of incarceration (short or long incarceration); and 2) Among people with short incarceration, high concentrations of MIP-3α were associated with a higher risk of a new TBI, and higher concentrations of Eotaxin was associated with a lower risk of a new TBI. Higher concentrations of sCD14 and TNF-α were associated with a higher risk of TB disease, and higher concentrations of IL-18 and MCP-1 were associated with a lower risk of TB disease.

In this study, immune mediators’ concentration varied among people newly infected by the time of incarceration. Levels of sCD14 and IL-18 were increased in individuals with short incarceration and IP-10 in those with long incarceration. These variations between people could occur for several reasons: 1) early exposure to *M. tuberculosis* can induce increased macrophage activation, leading to elevated sCD14 concentration. 2) Continuously exposed individuals (≥1 year in prison) could eliminate the bacteria repeatedly, owing to an effective immune response. 3) Other factors associated with prison entry include stress, physical activity, or dietary patterns. Several studies have documented changes in the production of pro-inflammatory cytokines when people are under a chronic stressful situation (25), variable levels of physical activity/exercise (26), or when a stress-generating situation is compounded by an infectious process, such as influenza vaccination (27). People deprived of liberty are exposed to acute and chronic stressors, given the social and safety conditions that prevail in prisons in which they live or the changes associated with freedom deprivation (28). In other words- the inflammatory milieu induced by environmental factors related to imprisonment may alter the immune response, thereby increasing the risk of MTB infection (29).

We found that increased circulating levels of MIP-3α were associated with a higher risk of having new TBI after short incarceration. The MIP-3α/CCL20 is a dendritic cell, T cell, B cell and monocyte chemoattractant involved in lymphocyte homeostasis and trafficking, cell proliferation and activation (21, 30, 31). Rivero-Lezcano et al. reported that the expression of MIP-3α increased up to 39-fold when monocytes from healthy donors were infected with *M. tuberculosis (* 32), and Lee et al. showed that MIP-3α was up-regulated in PBMC and bronchoalveolar lavage fluids from people diagnosed with TB compared to healthy controls after *in vitro* stimulation with the 30 kDa antigen (Ag) of *M. tuberculosis (* 33). MIP-3α concentration in serum/plasma samples is an attractive target for further evaluation in newly acquired MTB infection studies among people with documented recent exposure. In our research, PDL with a new TBI and with short incarceration had lower circulating levels of Eotaxin. Eotaxin has been reported as inhibited after *in vitro* infection by *M. tuberculosis (* 34), decreased in people diagnosed with TB disease, and with increasing levels after anti-TB treatment (35).

After adjusting for the other co-variables, individuals with elevated concentrations of MCP-1, a potent chemotactic factor for monocytes (36), had a lower risk of TB disease. In a model of macaques previously vaccinated with BCG, the production of β-chemokine MCP-1 increased in lung lesions of animals after five weeks of infection with MTB (37). These immune molecules are mainly associated with the recruitment of monocytes/macrophages, in keeping with the observation that animals had a higher frequency of macrophages (CD14+ CD68+) in peripheral blood three weeks post-infection with MTB (37). In this case, the increase in cells and β-chemokines may be a sign of the recruitment of these cells from peripheral blood to the site of infection in the lung. Our results show that the concentrations of MCP-1 were lower among the TB disease group compared to non-infected individuals; however, most reports about this chemokine have shown increased concentration among people diagnosed with TB (38, 39). Results may be different because of stimulation with mycobacterial antigens or the absence of stimulation in whole blood samples. Increased concentrations of MCP-1 have been reported in stimulated samples from individuals diagnosed with active TB, but higher concentrations in un-stimulated samples among non-infected individuals compared to individuals diagnosed with TB (40). Some additional explanations for those differences between the studies are the ancestry of the population, the specimen (serum, whole blood, or supernatants of cultured PBMC), and the presence of genetic polymorphisms regulating IL-18 or MCP-1 production (41) in the populations, which could alter the final structure, concentration, or function of the protein.

We also found that higher concentrations of sCD14 and TNF-α were associated with a higher risk of TB disease. These immune mediators are highly involved in monocyte/macrophage activation and trafficking pathways (42, 43). Macrophages are the main niche for the growth and survival of *M. tuberculosis (* 44); likewise, macrophages are the most important cells for infection control in animal and human models and critical cells in the host response during active disease (44–46) trying to limit the systemic spread of mycobacteria (47). Soluble CD14, whose membrane-bound portion is highly expressed in these cells, is a mediator of macrophage activation and serves as a receptor for mycobacterial lipoarabinomannan (43). It has been reported among people diagnosed with TB disease (48, 49), with or without concurrent HIV infection (47, 50), and individuals with diabetes mellitus (51). A published article by Lawn SD et al. suggests that sCD14 might increase the concentration of TNF-α due to the high load of mycobacterial antigens among people living with HIV and co-infected with TB disease (47). In our study, people diagnosed with TB had higher plasma concentrations of TNF-α, presumably contributing to cell recruitment, the production of other pro-inflammatory cytokines, and apoptosis of MTB-infected cells (52). Similar to our study, TNF-α has been reported to be increased in adults (49, 53–56) and children (57) diagnosed with TB disease.

Our findings show lower concentrations of IL-18 and MCP-1 among the TB group compared to non-infected individuals. Other researchers have shown that *M. tuberculosis*-stimulated culture supernatants from people diagnosed with TB have lower concentrations of IL-18 compared to those from healthy TST converters (58). IL-18 has an important function in TB as a pro-inflammatory cytokine (59); it plays an important role in the T-cell-helper type 1 (Th1) response, primarily by its ability to induce IFN-γ production in T cells and natural killer (NK) cells (60) and in combination with IL-12 triggers the antimicrobial protein cathelicidin and autophagy, resulting in inhibition of intracellular mycobacteria in macrophages and lung epithelial cells (61). Still, studies in humans are not conclusive. For example, Yamada G et al. (62) showed that increased serum IL-18 concentrations were associated with TB disease compared to healthy individuals (including TST converters).

Future studies are needed to validate and complement our cytokine and chemokine findings.

We did not find associations between IP-10, IL-17, IL-10, and a new TBI (the first one was not different between groups, and the latter two had concentrations below the lower detection limit), contrary to other publications (63, 64). This discrepancy may be attributed to the measurement of immune mediators after mycobacterial antigen and mitogen stimulation in prior studies (53, 56, 65–75), in contrast to the unstimulated measurements in our study. In the case of IL-17, there are six members in the IL-17 family, including IL-17A. IL-17B, IL-17C, IL-17D, IL-17E, and IL-17F (76, 77). Our study only measured Il-17A. Therefore, it is possible that other not measured subunits, such as IL-17F, were increased in plasma samples. In the same way, IL-17 is described as essential in the lung during recent infection (60). Still, perhaps blood sampling in the context of recent infection may have contributed to the reduced systemic level of IL-17.

Another reason for discrepant results may be that individuals included in other longitudinal studies were evaluated with only one TST administration, and those with a negative TST may represent a false negative result (61). In our previous studies, a second administration of TST identified an additional 11.6% positive TST individuals (78).

Our study’s most crucial distinguishing feature is the study design (cohort). In cross-sectional studies, the duration an individual has had TBI cannot be quantified. The main limitation of a cross-sectional approach is that human and primate studies have demonstrated there is a spectrum of TB stages (5, 6, 79, 80), on which there is little published work (4, 6), and that potentially could alter the concentration of the immune parameters according to the stage of the infection. The cohort design allowed us to quantify the concentration of cytokines at a temporal point close to the time of infection (new infection) and identify individuals in whom infection occurred recently. Borgstrom WE et al., using mathematical models and CD4+ T-cell flow-cytometry data, showed that the most specific prediction of recent TBI was a high proliferative CD4+ response to CFP-10 and PPD, and a low response to ESAT-6 at ≤1 month after exposure (81).

Our study has other strengths: rigorous selection criteria among people diagnosed with TB, including only those with microbiological confirmation and with less than 15 days of treatment, rule out of booster effect, and rigorous monitoring and follow-up every three months to all uninfected participants.

The main limitation of this study is that some bacterial factors may modify the response to cytokines, such as the virulence of some strains; however, in people with TBI, it is not feasible to isolate the mycobacterium, and therefore the role of MTB strain cannot be assessed yet.

## 5 Conclusion

Our study found that immune markers vary according to the time of incarceration. Among people with short incarceration, high concentrations of MIP-3α were associated with a higher risk of a new TBI, and higher concentrations of Eotaxin were associated with a lower risk of a new TBI. Higher concentrations of sCD14 and TNF-α were associated with a higher risk of TB disease, and higher concentrations of IL-18 and MCP-1 were associated with a lower risk of TB disease. It is necessary to have more cohort studies that evaluate the changes over time of these and other cytokines/chemokines to understand the immune mechanisms across the spectrum of TB.

## Data availability statement

The raw data supporting the conclusions of this article will be made available by the authors, without undue reservation.

## Ethics statement

The studies involving human participants were reviewed and approved by Ethics Committees of the Universidad Pontificia Bolivariana and the University of Manitoba. The patients/participants provided their written informed consent to participate in this study.

## Author contributions

Substantial contributions to the conception or design of the work: MH, YK, ZR. Acquisition of data: MH. Analysis and interpretation of data: MH, YK, LL, DM, ZR. Drafting the article and revising it critically for important intellectual content: MH, YK, LV, PM, ZR. Final approval of the version to be published: MH, YK, LV, LL, DM, PM, ZR. Agreement to be accountable for all aspects of the work in ensuring that questions related to the accuracy or integrity of any part of the work are appropriately investigated and resolved: MH, YK, LL, DM, LV, PM, ZR. Principal investigator and funding acquisition: ZVR. All authors contributed to the article and approved the submitted version.
