# Supplementary material for: Cytokine/chemokine profiles in people with recent infection by Mycobacterium tuberculosis
Source: Front Immunol. 2023 May 16;14:1129398. doi: 10.3389/fimmu.2023.1129398 (PMC10229054; doi:10.3389/fimmu.2023.1129398)
Supplement: Supplementary file 1 [file DataSheet_1.docx]

**Supplementary 1**. Selection criteria and definitions used during the cohort study. Sep 2016-dec 2018, Colombia.

The information about the prisons (capacity), the cohort ensemble (people screened, number of TST applications, number of TST positive at baseline, etc.) and the participants (number of people deprived of liberty -PDL, Tuberculin skin test-TST- negative), has been published by Herrera M *et al*. [1].

**Population screened and participants**: We screened 540 males from two Colombian prisons. We used two-step TST for new tuberculosis infection (TBI) screening in 386 of them at baseline. We read the TST in 358/386 participants, and 210 tested positive. The cohort started with 124 participants (two-step TST negative), and during the follow-ups, 25 PDL tested positive in the TST.

**Inclusion criteria**.

Individuals who had a negative two-step TST at baseline, who agreed to participate in the study and signed a written consent form.

**Exclusion criteria.**

1) Administration of live vaccines (measles, mumps, and rubella; varicella; or the live attenuated

influenza vaccine) in the last four weeks before TST administration, 2) severe adverse reactions with a previous TST administration, 3) previous active TB, and 4) people who had a sentence of less than two years of incarceration in prison (released or moved into house detention) because of the challenges for follow up.

**TST procedure and TBI diagnosis**

The TST (tuberculin purified protein derivative RT-23, 2 tuberculin units/ 0.1 ml, (Statens Serum Institute, Copenhagen, Denmark) was administered according to US Centers for Disease Control and Prevention (CDC) recommendations[2]. Reading was performed within 48–72 h of administration and measured and recorded in mm induration. A second TST was applied in the case of a negative result in the first application to detect the booster effect. A new TST was administered every six months after the recruitment according to CDC recommendations. Reading was performed by experienced research nurses 48 or 72 h after TST administration and measured in millimetres (mm) induration.

**Follow-up.**

We administered the TST every six months for up two years or until a positive TST result.

**Outcome**: Conversion (new TBI) was defined as a new TST reaction (after booster application) ≥10 mm and an increase of at least 6 mm[3].

### Diagnosis of pulmonary TB

We included active TB cases from the same prisons during the cohort follow-up. Individuals with a sputum smear-positive provided a spontaneous sputum sample for microbiological confirmation of *Mycobacterium tuberculosis*.  All the sputum samples were processed using the conventional sodium hydroxide-N-acetyl-L-cysteine method, with standard decontamination and concentration methods. A smear was prepared for auramine-rhodamine staining to visualize acid-fast bacilli (AFB). The sputum sample was inoculated in Lowenstein-Jensen (LJ) medium, in a mycobacterial growth indicator tube (MGIT) incubated in MGIT 960 BACTEC instrument (BD Diagnostics, Sparks, MD, USA), and in thin-layer agar (TLA) for the detection of resistance to rifampicin and isoniazid as previously reported[4]. *M. tuberculosis* was identified by standard biochemical tests.

**REFERENCES**

1. Herrera M, Keynan Y, López L, Marín D, Arroyave L, Arbeláez MP, et al. Incidence and Risk Factors Associated with Latent Tuberculosis Infection and Pulmonary Tuberculosis among People Deprived of Liberty in Colombian Prisons. Am J Trop Med Hyg. 2021 Dec 6;

2. CDC | TB | Hojas informativas - Pruebas de tuberculosis [Internet]. 2021 [cited 2021 Nov 22]. Available from: https://www.cdc.gov/tb/esp/publications/factsheets/testing/skintesting_es.htm

3. Menzies D. Interpretation of repeated tuberculin tests. Boosting, conversion, and reversion. Am J Respir Crit Care Med. 1999 Jan;159(1):15–21.

4. Rueda ZV, López L, Vélez LA, Marín D, Giraldo MR, Pulido H, et al. High Incidence of Tuberculosis, Low Sensitivity of Current Diagnostic Scheme and Prolonged Culture Positivity in Four Colombian Prisons. A Cohort Study. Dowdy DW, editor. PLoS ONE. 2013 Nov 21;8(11):e80592.
