# Supplementary material for: Cytokine/chemokine profiles in people with recent infection by Mycobacterium tuberculosis
Source: Front Immunol. 2023 May 16;14:1129398. doi: 10.3389/fimmu.2023.1129398 (PMC10229054; doi:10.3389/fimmu.2023.1129398)
Supplement: Supplementary file 2 [file DataSheet_2.docx]

**S2.** Cytokines/chemokines concentrations in new TBI at baseline, pre-conversion, and conversion time, by the time of incarceration

| **Cytokines/**  **Chemokines** | **Measurements at baseline, pg/ml median (IQR)** | |  | **Measurements at TST conversion, pg/ml median (IQR)** | | |
| --- | --- | --- | --- | --- | --- | --- |
|  | ≥**1 year, n= 9** | ≤**3 months, n= 12** | ***p**** | ≥**1 year** (pg/ml)**, n= 8** | ≤**3 months, n= 11** | ***p**** |
| sCD14 (ng/ml) | 2181.6 (1997.5-2406.9) | 2764.2 (2412.9-3339.6) | **0.0230** | 1984.8 (1383.4-2376.2) | 2621.1 (2191.3-4023.9) | **0.0352** |
| MIP-3α | 12.9 (7.5-20.8) | 17.9(13.6-21.9) | 0.1768 | 20.4 (9.9-28.5) | 16.7 (13.6-22.5) | 0.8688 |
| IL-18 | 79.9 (45.8-83.0) | 144.4 (95.2-193.7) | **0.0330** | 73.0 (50.3-90.5) | 187.5 (88.4-200.4) | **0.0105** |
| Eotaxin | 245.7 (109.2-349.9) | 158.7 (58.8-305.7) | 0.4773 | 228.9 (142.2-464.8) | 121.6 (59.8-356.8) | 0.1864 |
| INF-γ | 0.05 (0.05-1.01) | 0.25 (0.05-0.7) | 0.9115 | 0.2 (0.05-1.38) | 0.25 (0.05-0.61) | 0.9325 |
| MIP-1β | 16.4 (7.1-18.4) | 11.7 (8.7-15.0) | 0.4344 | 10.1 (7.4-16.1) | 13.3 (8.9-17.4) | 0.5081 |
| TNF-α | 5.0 (4.4-7.2) | 4.2 (3.2-5.4) | 0.1178 | 5.4 (3.7-7.1) | 3.9 (3.5-5.8) | 0.4088 |
| IP-10 | 310.7 (240.0-405.8) | 205.5 (172.7-250.1) | 0.1179 | 211.4 (192.4-252.9) | 229.1 (138.7-269.3) | 0.5633 |
| MCP-1 | 217.3 (181.9-285.4) | 210.1 (174.7-264.5) | 0.9433 | 280.1 (235.3-345.5) | 247.2 (193.1-304.6) | 0.4090 |

| **Cytokines/**  **Chemokines** | **Measurements 3 months before TST conversion, pg/ml median (IQR)** | | |
| --- | --- | --- | --- |
|  | ≥**1 year, n= 7** | ≤**3 months, n= 11** | ***p**** |
| sCD14 (ng/ml) | 1915.0 (1563.3-2175.2) | 2622.4 (2054.3-2903.5) | **0.0164** |
| MIP-3α | 7.5 (1.21-34.9) | 16.2 (14.7-24.8) | 0.2571 |
| IL-18 | 60.3 (39.5-112.9) | 154.2 (80.3-198.4) | **0.0333** |
| Eotaxin | 195.7 (112.2-697.8) | 119.5 (47.5-310.1) | 0.2215 |
| INF-γ | 0.05 (0.05-0.9) | 0.06 (0.05-0.5) | 0.6908 |
| MIP-1β | 11.5 (9.7-19.8) | 11.7 (8.2-16.1) | 0.4968 |
| TNF-α | 7.9 (3.2-10.2) | 3.7 (2.9-4.3) | 0.1128 |
| IP-10 | 243.2 (198.3-332.4) | 128.5 (108.1-185.8) | **0.0075** |
| MCP-1 | 240.7 (228.3-279.5) | 242.1 (201.2-316.5) | 0.6836 |

IQR: interquartile range; TBI: tuberculosis infection **p* value using Mann-Whitney U test. New TBI [people with negative two-step TST that became positive during follow-up were divided into TBI with sort incarceration (they had ≤3 months of incarceration at enrolment) and TBI with long incarceration (they had ≥1 year of incarceration at enrolment)]. Values are reported in pg/ml for all cytokines/chemokines except for sCD14 which is in ng/ml. Variables with statistical significance are shown in bold.
